# The Anti-Vivisection Movement

**Published:** 1900-02

**Authors:** 


### THE ANTI-VIVISECTION MOVEMENT.

IT would be redundant to call attention to the vast benefit to humanity afforded by physiological experimentation upon lower animals, but it does seem necessary to occasionally remind the public that medicine is essentially a humane calling and that the devotees of its art are the militants of mercy. Their constant effort is directed to the relief of pain and the production of insensibility to it, yet they are held up as monsters of brutality who know no higher pleasure than the sight of the red track of the knife and who hear a lullaby in the cry of anguish. Even Tennyson was not above depicting a surgeon in this light, but be it said that his rhyme is little better than doggerel, and shows evidence of senile lachrymoseness, and he assuredly would not have put forth such flatulent drivel in his days of mental compactness. Nobody seriously believes that doctors as a class are cruel, and, by an amplification of this belief, if they are not cruel to man they are not cruel to animals, for cruelty is a consistent quality.

Nothing can be more shortsighted than the endeavors of the anti-vivisectionists, and nothing would more surely react upon them for harm than the suppression of physiological experiment upon the living organism. They are blind to the purpose of laboratory work, and they see in it only mutilation of man's patient friends. The belief is founded upon no more substantial base than dense ignorance. They belong to the generation of them who having eyes see not, and having ears hear not, for who is so blind and so deaf as he who *will* not see or hear. Would it not be better for these myopic, sour-visaged fanatics to turn their seething energies to the suppression of the game and fish hog who is depopulating our streams and forests, and of the slaughter of animals and birds for their fur and plumage which is going on year by year to supply the Moloch of fashion. They know the suppression of vanity is a larger order than attacking vivisection. "Since our women must walk gay and money buys their gear," they allow Ephraim unmolested to return to as many idols as he wishes, but they have tried and are to try again to legislate out of existence one of the principal factors contributing to the tenableness of this infected old world and to the health and happiness of its inhabitants.

It is sincerely hoped that every scientific worker will use his influence to prevent the passage of the Gallinger bill, referred to *in extenso* in a communication from Dr. Keen in the January issue of THE JOURNAL-RECORD. W.

